# Gender Differences in Schizophrenia and First-Episode Psychosis: A Comprehensive Literature Review

**DOI:** 10.1155/2012/916198

**Published:** 2012-04-08

**Authors:** Susana Ochoa, Judith Usall, Jesús Cobo, Xavier Labad, Jayashri Kulkarni

**Affiliations:** ^1^Research and Developmental Unit of Parc Sanitari Sant Joan de Déu, CIBERSAM. GTRDSM, Sant Boi de Llobregat, 08330 Barcelona, Spain; ^2^Department of Mental Health, Corporació Parc Sanitari Taulí, GTRDSM, Sabadell, 08830 Barcelona, Spain; ^3^Department of Mental Health, Institut de Psiquiatria Pere Mata, GTRDSM, Reus, Tarragona, Spain; ^4^Monash Alfred Psychiatry Research Centre (MAPrc), “We Mend Minds,” Old Baker Building, The Alfred Commercial Road, Melbourne, VIC 3004, Australia

## Abstract

Recent studies have begun to look at gender differences in schizophrenia and first-episode psychosis in an attempt to explain the heterogeneity of the illness. However, a number of uncertainties remain. This paper tries to summarize the most important findings in gender differences in schizophrenia and first-psychosis episodes. Several studies indicate that the incidence of schizophrenia is higher in men. Most of the studies found the age of onset to be earlier in men than in women. Findings on symptoms are less conclusive, with some authors suggesting that men suffer more negative symptoms while women have more affective symptoms. Premorbid functioning and social functioning seem to be better in females than males. However, cognitive functioning remains an issue, with lack of consensus on differences in neuropsychological profile between women and men. Substance abuse is more common in men than women with schizophrenia and first-episode psychosis. In terms of the disease course, women have better remission and lower relapse rates. Lastly, there is no evidence of specific gender differences in familial risk and obstetric complications. Overall, gender differences have been found in a number of variables, and further study in this area could help provide useful information with a view to improving our care of these patients.

## 1. Introduction

Schizophrenia and first-episode psychosis are disorders with considerable heterogeneity in several of its basic features. There is great variability in clinical presentation, disease course, and response to both pharmacological and psychosocial treatment. Some aspects of this heterogeneity may be gender related and, given the reliability, stability, and validity of its definition, study of the gender variable may help explain the differences. Gender differences have been studied extensively in recent decades and although there are definite findings, much uncertainty remains about the extent of the differences. This paper will try to summarize the most relevant research done around the world on gender differences in schizophrenia and first-psychosis episodes. The topics discussed in this paper will be prevalence and incidence, age of onset, symptoms, premorbid, social and cognitive functioning, substance abuse, course of illness, physical health and metabolic complications, and familial risk and obstetric complications. The paper will try to assess gender differences studies on each of these topics in people with schizophrenia and in first-episode psychosis. A greater understanding of the gender differences presents in schizophrenia and first-episode psychosis can help us design more effective preventive and intervention actions.

## 2. Prevalence and Incidence of Schizophrenia

The existence of gender differences in the incidence of schizophrenia has been subject to debate. Traditionally, it was accepted that the incidence and prevalence of schizophrenia was the same in men and women [1]; however, recent studies suggest gender differences in the incidence of the illness. Lewine et al. [2] were the first to note that by using more restrictive criteria for the diagnosis of schizophrenia, the number of women excluded from the definition is greater than men. Castle et al. [3] applied a different set of diagnostic criteria to an incidence sample of patients with a wide range of nonaffective psychosis presentations and found that the effect of different diagnostic criteria on the gender ratio is profound. For example, the Feighner restrictive criteria found a ratio of female-men 0.41 : 1 and the ICD the ratio female-men is 0.92 : 1. Using standard diagnostic criteria in an incidence population study, a meta-analysis by Aleman et al. [4] confirmed that the men had a higher incidence (ratio 1.42). However, the recent studies of prevalence of schizophrenia in general population did not find gender differences [5, 6]. One possible explanation for the disparity between incidence and prevalence could be related to compliance with treatment and higher rates of suicide completion in men than in women [7]. Another possible explanation could be related to the design of the studies, for example, one centered more in epidemiological resources and the incidence was centered in clinical data.

No gender differences have been found in prevalence of schizophrenia in epidemiological studies; however, it seems that more new cases of schizophrenia have been detected in men.

## 3. Age of Onset

Differences in age of onset are the most replicated finding in studies into gender differences in schizophrenia. [8–10] Men usually develop the illness at age 18–25, while in women, the mean age of onset is 25–35. Furthermore, the onset distribution curves for males and females are not isomorphic. Women seem to have two peaks in the age of onset of disease: the first after menarche and the second once they are over 40 [3, 11]. However, in 1998, Castle et al. found that early-onset age distribution is similar between men and women [12]. The major prevalence of women once they are over 40 years could be explained by the reduction of estrogens after menopause according the estrogenic hypothesis of schizophrenia [13]. However, a number of studies found no gender difference in the age of onset [14–16].

Some authors have suggested that differences in age of onset appear to depend on the presence or absence of family history, with no differences being found between men and women if they had a family history [17, 18].

Besides, the findings of early age of onset in men have been replicated in first-episode psychosis [19, 20], indicating a consistency with the results found in schizophrenia.

Gender differences have been found in most of the studies done in age of onset in schizophrenia and first-episode psychosis, showing a different profile of onset of illness between women and men.

## 4. Symptoms

The study of gender differences in symptoms of schizophrenia has been one of the most explored issues. However, the results in this area are inconclusive.

Several studies have found gender differences in negative symptoms, showing that in males, they were more severe [21–23]. Moreover, in a sample of 276 people with schizophrenia, Galderisi et al. [10] found that men scored higher in disorganization and negative symptoms. In a large sample of patients with psychosis, Morgan et al. [24] identified a higher prevalence of depressive symptoms and lower prevalence of negative symptoms in women. Higher prevalence of depressive and anxiety symptoms in women had been found in previous studies [3, 25].

Nevertheless, most of the studies [15, 26, 27] found no significant clinical differences in symptoms, [19, 26, 27] which is in line with our team's findings [28].

Results from the assessment of symptoms in first-episode psychosis are also inconclusive. In a group of patients with schizophrenia admitted for the first time, Szymanski et al. [28] found that women presented more anxiety, illogical thinking, inappropriate affect, and bizarre behavior than men. Cotton et al. [29] found that women presented higher levels of affective symptoms than men. However, no gender differences were found in the study by Barajas et al. [30].

In relation to diagnosis, Andia et al. [31] found a higher percentage of women diagnosed with paranoid schizophrenia.

There is not a clear influence of gender in the symptoms presented in people with schizophrenia and first-episode psychosis. However, the studies that found gender differences describe higher presence of negative and disorganization symptoms in men and higher prevalence of affective symptoms in women.

## 5. Premorbid Functioning

Better premorbid functioning has been associated with a better prognosis for the illness. Gender differences here could, therefore, have a bearing on how the schizophrenia evolves. In general, most studies have found gender differences in premorbid functioning, this being worse in men than in women [21, 24, 32–34]. McGlashan and Bardenstein [32] found that women had better premorbid social functioning and marital adjustment. However, in a sample of 113 patients, females and those with a diagnosis of schizoaffective disorder had better premorbid adjustment in the academic domain, but not in the social domain [34].

These results have been replicated in a sample of first-episode psychosis [35, 36]. Little is known about gender differences in the psychosis prodromes. In adolescents at ultra-high-risk (UHR) of imminent onset of psychosis, being female was a significant predictor of conversion to affective psychosis two years after ascertainment [36] and young male adults with a diagnosis of schizotypal disorder had a fourfold risk of conversion to schizophrenia one-year after enrolment when compared to females [36].

In a study by Rachel Willhite with sixty-eight ultra-high-risk patients in California (USA), the authors investigated gender differences in symptoms, functioning, and social support. There were no gender differences in demographic variables, symptoms, or functioning at baseline. Males were found to have significantly higher levels of negative symptoms and marginally lower levels of functioning and females reported higher levels of social support at baseline. Differences in negative symptoms were found to mediate differences in functioning between male and female patients. This study suggests that gender-based differences in symptom presentation and functional outcome may predate conversion to psychosis [37].

Also interesting is the association found between worse premorbid adjustment, insidious onset, and negative symptoms [33, 38]. Thus, one explanation for the worse premorbid functioning in men could be the earlier age of onset.

Most of the literature that assesses premorbid functioning found that women have higher levels of premorbid adjustment and reported higher levels of social support than men.

## 6. Social Functioning

In general, studies that have examined gender differences in social functioning have found better performance in women. Chaves et al. [38] found that women were better adapted and presented less disability than men. In a three-year follow-up study of 86 patients who had a first episode of schizophrenia, using the DAS scale, Vázquez-Barquero et al. [39] found that men had a worse prognosis. A previous study by our group of 239 patients with schizophrenia living in the community also found that men scored higher in disability (measured with the DAS scale) [40, 41]. Vila-Rodriguez et al. [42] replicated these results, finding that women scored higher in social functioning assessed by LSP. Recently, following a 20-year longitudinal study, Grossman et al. [43] found that women had better global functioning over the course of the illness.

However, after assessing the occupation rate and several psychosocial functioning indices (PSP, PSRS, and UPSA-B) in patients with schizophrenia, Galderisi et al. [10] found no gender differences in social outcome. Additionally, in a long-term study (15 years), Bottlender et al. [44] failed to detect gender differences in social disability in patients with schizophrenia, schizoaffective, and affective disorders through the DAS.

In first-episode psychosis, the results obtained by Cotton et al. [29] show that women had higher levels of functioning (assessed by GAF, unemployment index and living with family).

In relation to stressful life events, several studies have found that females need more exposure to stressful life events than males to trigger a psychotic disorder [45, 46]. It seems that women with schizophrenia presented higher resilience than men to cope with stress situations and women need higher risk factors in order to develop a psychosis than men do.

Regarding the needs of patients with schizophrenia, men presented more basic (accommodation, food, daily activities) and functional needs (education, money, personal care), while women scored higher in the prevalence of service needs (information about the illness, benefits, transport) [47]. This finding indicates that women perform better in basic and functional domains than men, and men should be trained in order to acquire these functional skills.

In relation to fertility, some reports suggest reduced procreation among men with schizophrenia, but the cause is unknown. Male cases were significantly more likely than female cases to be single and childless [48]. In contrast, the Indo-US Project on Schizophrenia Genetics detected that a reproductive deficit observed among US males was not observed among the Indian men. Conjugal status was a significant covariate for reproduction in both samples. The reproductive deficit may be due to difficulties in establishing long-term conjugal relationships among the US men and not in the Indian sample. According to the authors, the differences may also reflect underlying cultural variations related to marital practices [49].

Women with schizophrenia and first-episode psychosis performed better in social functioning according to objective assessments (fertility, being married) and social scales assessment. Moreover, women presented less basic and functional needs and need more exposure to life events in order to develop a psychosis illness.

## 7. Cognitive Functioning

Gender differences in cognitive domains have been another controversial issue. A number of authors have demonstrated that men score worse in attention, language, and executive function than women [50–53]. Vaskinn et al. [54] suggest better functioning in neuropsychological performance in women than men, except in the category of attention. Bozikas et al. [55] found that women performed better than men in verbal learning and memory.

Bilder et al. [56] found that males performed better in the information subtest of the WAIS, while women performed better in the Digit Symbol subtest. Other studies have shown cognitive functioning to be worse in women with schizophrenia than men [57, 58].

In the study by Karilampi et al. [59], better cognitive function was predicted by higher psychosocial functioning levels in males but by lower symptom levels in females, suggesting a slight difference between women and men in the domains relating to cognitive function, which need to be taken into account.

Other studies, however, found no gender difference in the assessment of cognitive domains [60–62].

Gender differences in cognitive function in people with schizophrenia remained controversial. The studies that found gender differences indicate higher levels of functioning in women especially in the language, executive, and memory domains.

## 8. Substance Abuse

Substance abuse presented a higher prevalence in people with schizophrenia and first-episode psychosis [63–65]. The rates indicated that men consume more cannabis than women [64, 66]. In the case of first-episode psychosis, men had a higher prevalence of cannabis use than women [29, 64, 66, 67]. Moreover, Rodríguez-Jiménez et al. [68] found that men have higher comorbidity of cocaine and hallucinogen use and of cannabis use than women. In the case of alcohol abuse, the data show that males present higher levels of consumption than women [10].

In addition, Arendt et al. [69] demonstrate that the risk of developing psychosis is higher in men who consume cannabis than women. The study assessed a total of 535 people with a cannabis-induced psychosis over three years, and the rates for developing schizophrenia were 47.6% in males versus 29.8% in women.

Men presented higher prevalence of substance abuse and higher levels of comorbidity than women. Moreover, it seems that substance abuse could be a risk factor for developing psychosis in males.

## 9. The Course of the Illness

The disease course for schizophrenia has been reported as following different patterns in males and females. Uggerby et al. [70] studied the prevalence of institutionalized and noninstitutionalized people with schizophrenia in Denmark in a sample of 22,395 people. The results showed that being male was one of the predictors of institutionalization. Gender has also been identified as one of the factors influencing clinical remission, with relapse rates being higher in men and remission rates higher in women [71].

With regard to hospitalizations, Usall et al. [40] found that the number of previous hospitalizations was similar for both men and women. However, women required less time in hospital than men at baseline. After a three-year followup of these patients, the results indicated that women had fewer admissions than men and the length of stay was shorter (men, 40 days versus women, 5.8) [72]. In the SOHO Study, however, Haro et al. [71] found that women presented a higher risk of hospitalization than men.

The efficacy and tolerance of the different antipsychotic treatments may be gender-sensitive. Most studies found that women respond better to typical antipsychotics [28, 73] and olanzapine [74, 75] The results for clozapine are more controversial [75–77]; as for risperidone, the few studies in this direction have not found differences [78]. Premenopausal women had a significantly better treatment response to olanzapine than postmenopausal women, regardless of chronicity and treatment [74].

No significant gender differences were found, either in treatment response or neurological side effects, in patients treated with risperidone [78]. There were, however, some concerns about parkinsonian symptoms with atypical neuroleptics being more frequent in women [79].

The results found regarding course of illness are controversial; however, it seems that women presented higher rates of remission, less days of hospitalization, and better response to typical antipsychotics than men.

## 10. Physical Health and Metabolic Complications

There have also been concerns about gender differences in relation to physical health and metabolic complications in psychosis.

The metabolic impact of antipsychotic treatments in women (and men) is significant. Atypical and older antipsychotics are very useful drugs, but they can be associated with hyperprolactinemia and related disorders. These endocrine aspects are particularly significant. Women have greater metabolic and endocrine-induced antipsychotic side effects. In fact, every woman exposed to atypical antipsychotics is at risk of developing hyperprolactinemia-related problems, particularly young women [80–82]. Previous studies have consistently reported a higher prevalence of hyperprolactinemia in women receiving antipsychotics, and cross-sectional studies in the USA and UK have estimated hyperprolactinemia prevalence rates of up to 42% in men and 75% in women with schizophrenia who were receiving conventional antipsychotics or risperidone [83, 84]. It is known that hyperprolactinemia is associated with a number of physical health problems in males and females, particularly endocrine and immunological system changes, as well as growth hormone alterations. For example, in one study which included 150 women, 14% were observed to develop galactorrhea within 75 days of initiating treatment with conventional antipsychotics [85]. Hyperprolactinemia affects long-term health in women. Menstrual irregularities have been found in up to 48% of women receiving antipsychotic treatment [80, 82]. Reduced bone mineral density has been demonstrated in 57% of men and 32% of women treated with prolactin-raising antipsychotics for over 10 years [86]. One case-control study investigated whether potential treatment-emergent decreases in bone mineral density could confer an increased risk of hip fractures in patients with a history of schizophrenia [87].

Although sexual dysfunction appears to be inherent to the illness in patients with schizophrenia, it is also frequently reported during antipsychotic treatment, with interesting gender differences. More than 50% of males and 30% of females have been shown to experience sexual dysfunction during conventional antipsychotic treatment. This specific secondary effect may be relevant to adherence in some patients [88, 89].

A Spanish national cross-sectional study in 733 patients diagnosed with schizophrenia on treatment with second generation antipsychotics and admitted to short-stay hospital units detected different cardiovascular risk factors in women than in men. Men were treated for hypertension (OR = 25.34, *P* < 0.03) and women for diabetes (OR = 0.02, *P* < 0.03) [90].

Metabolic syndrome is associated with the development of coronary heart disease and diabetes mellitus. A higher presence of metabolic syndrome has been detected in females. In a Turkish sample, Boke et al. found that 61.4% of females, but only 22.4% of males, had metabolic syndrome [91].

The prevalence of metabolic syndrome in 1460 US patients from the Clinical Antipsychotic Trials of Intervention Effectiveness (CATIE) showed important gender differences. In females, depending on the criteria used, it was 51.6% (NCEP) or 54.2% (AHA), compared to 36.0% (NCEP; *P* = 0.0002) or 36.6% (AHA; *P* = 0.0003) for males. 73.4% of all females (including nonfasting subjects) met the waist circumference criterion compared to 36.6% of males. In a logistic regression model with age, race, and ethnicity as covariates, CATIE males were 138% more likely to have metabolic syndrome than a general population-matched sample (NHANES), and CATIE females, 251% more likely than their general-population counterparts. Even when controlling for differences in body mass index, CATIE males were still 85% more likely to have MS than the NHANES male sample and CATIE females, 137% more likely to have MS than females in NHANES [92].

In contrast, in a study devoted to detect coronary heart disease risk and prevalence of metabolic syndrome in 268 patients with schizoaffective disorder receiving antipsychotics, the authors detected no gender differences, but coronary heart disease risk and prevalence of metabolic syndrome were higher among patients with schizoaffective disorder. The prevalence of metabolic syndrome was associated with age and severity of disease, but not with gender [93].

Individuals with nonaffective psychosis appear to have an increased prevalence of abnormal glucose tolerance prior to antipsychotic treatment, but this predisposition also appears not to be gender sensitive [94]. Similarly, a large community study in Ontario (Canada) with 1123 schizophrenic outpatients failed to detect gender differences in dysglycemia [95].

Regarding metabolic and endocrine-induced antipsychotic side effect, women presented higher prevalence of symptoms. Hyperprolactinemia and diabetes are more present in women, while hypertension is more prevalent in men with schizophrenia.

## 11. Familial Risk and Obstetric Complications

Various studies have found a higher risk of schizophrenia in relatives of women than in relatives of men [96–98].

However, Kendler and Walsh found no gender differences in the familial risk of schizophrenia [99]. These authors studied familial risk in a sample of 354 first-degree relatives of patients with schizophrenia from the Roscommon Family Study who were interviewed personally. It also explored the possible association between age at onset, gender, and familial risk. The results of Pulver and Liang [97] showed that relatives of men with schizophrenia who have an age of onset under 17 have a significantly higher risk of schizophrenia. However, the authors also found an association between age at onset of schizophrenia and familial risk in women. Other studies have found no interaction between age of onset, gender, and familial risk [100].

Results on whether gender differences exist in the incidence of obstetric complications in patients who will develop schizophrenia have been inconsistent. Some studies have found more obstetric complications in men [101, 102]. However, other studies have found no gender differences [103, 104] and others still have found more obstetric complications in women [105]. The influence of gender in the prevalence of obstetric complications, therefore, remains unclear.

Women need higher presence of familial risk than men in order to develop the illness. However, there are not clear results about the influence of gender in the number of obstetric complications.

## 12. Conclusions

In conclusion, although the extent of gender differences in schizophrenia and first-episode psychosis is a controversial issue, this paper discusses some of the most replicated gender differences in schizophrenia and first-episode psychosis. Several studies indicate that schizophrenia and first-episode psychosis are less incident in women than in men but, in the case of women, it seems that the prognosis of the illness, the social functioning and the response to treatment is better. According to most of the studies revised, one possible explanation of this better adjustment could be that women presented a higher age of onset than men, which allows them to adjust better to the requirements of the community. The estrogen hypothesis tries to explain why women have a later age of the onset. According to this hypothesis some therapeutic treatments associated with estrogens could be useful for improving symptoms and cognition, especially in women. Moreover, the review shows us that women need more risk factors in order to develop schizophrenia than men (more familial risk, more presence of life events). This findings are in agreement with the neurodevelopment hypothesis, where men seem to present a more deteriorated profile than women before the onset of the illness.

One of the limitations of this paper is that, given the breadth of the subject, some issues have not been commented. Social influence of the context and the fact that most of the studies have been done in developed countries is a clear limitation that should be taken into account in the future.

From the reviewed literature, we conclude that women with schizophrenia perform better in several areas than men; however, future research should be addressed to study gender differences to clarify the remaining controversial issues. Novel sex-specific treatments could be developed to better meet the needs of people with schizophrenia and first-episode psychosis.
